# Diabetes and aortic dissection: unraveling the role of 3-hydroxybutyrate through mendelian randomization

**DOI:** 10.1186/s12933-024-02266-3

**Published:** 2024-05-07

**Authors:** Shi Qiu, Zhen Liu, Wei-Dong Jiang, Jin-Hui Sun, Zeng-Qiang Liu, Xiao-Di Sun, Chun-Ting Wang, Wen Liu

**Affiliations:** 1 Department of Cardiac Surgery, The Second Hospital of Shandong University, 250033 Jinan, Shandong China; 2 Department of Cadre Health Care, The Second Hospital of Shandong University, 247 Beiyuan Street, 250033 Jinan, Shangdong People’s Republic of China

**Keywords:** Diabetes, 3-hydroxybutyrate, Dissection of aorta, Genome-wide association study (GWAS), Mendelian randomization (MR)

## Abstract

**Background:**

In observational and experimental studies, diabetes has been reported as a protective factor for aortic dissection. 3-Hydroxybutyrate, a key constituent of ketone bodies, has been found to favor improvements in cardiovascular disease. However, whether the protective effect of diabetes on aortic dissection is mediated by 3-hydroxybutyrate is unclear. We aimed to investigate the causal effects of diabetes on the risk of aortic dissection and the mediating role of 3-hydroxybutyrate in them through two-step Mendelian randomization.

**Materials and methods:**

We performed a two-step Mendelian randomization to investigate the causal connections between diabetes, 3-hydroxybutyrate, and aortic dissection and calculate the mediating effect of 3-hydroxybutyrate. Publicly accessible data for Type 1 diabetes, Type 2 diabetes, dissection of aorta and 3-hydroxybutyrate were obtained from genome-wide association studies. The association between Type 1 diabetes and dissection of aorta, the association between Type 2 diabetes and dissection of aorta, and mediation effect of 3-hydroxybutyrate were carried out separately.

**Results:**

The IVW method showed that Type 1 diabetes was negatively associated with the risk of aortic dissection (OR 0.912, 95% CI 0.836–0.995), The weighted median, simple mode and weighted mode method showed consistent results. The mediated proportion of 3-hydroxybutyrate on the relationship between Type 1 diabetes and dissection of aorta was 24.80% (95% CI 5.12–44.47%). The IVW method showed that Type 2 diabetes was negatively associated with the risk of aortic dissection (OR 0.763, 95% CI 0.607–0.960), The weighted median, simple mode and weighted mode method showed consistent results. 3-Hydroxybutyrate does not have causal mediation effect on the relationship between Type 2 diabetes and dissection of aorta.

**Conclusion:**

Mendelian randomization study revealed diabetes as a protective factor for dissection of aorta. The protective effect of type 1 diabetes on aortic dissection was partially mediated by 3-hydroxybutyrate, but type 2 diabetes was not 3-hydroxybutyrate mediated.

**Graphical abstract:**

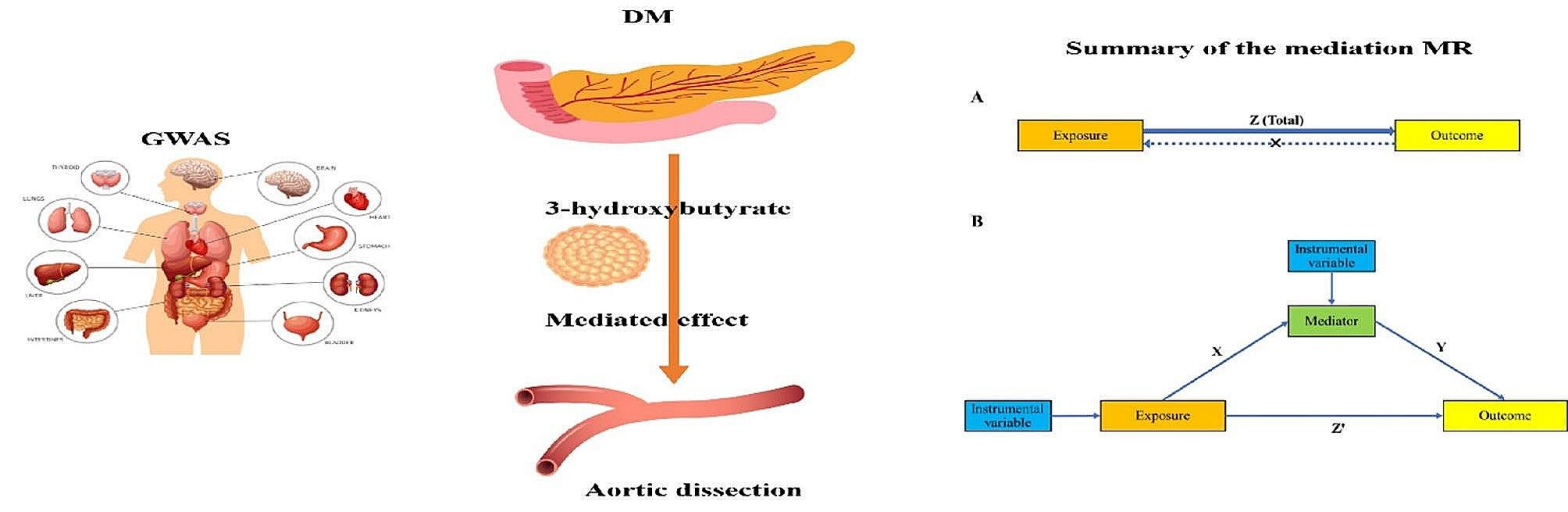

## Introduction

Aortic dissection is a severe cardiovascular condition characterized by the destabilization of the aortic wall [1], resulting in a sudden onset of symptoms such as severe pain radiating to the back and chest [2]. The acute phase of aortic dissection is characterized by the development of a tear in the aortic wall, leading to the formation of a false lumen. This process may be accompanied by immediate aortic enlargement or progressive dilation over time [3]. Left untreated, aortic dissections, particularly type A dissections, can lead to mortality rates as high as 90% within 30 days [4]. Despite notable progress in surgical interventions for aortic dissections, acute type A aortic dissection remains a significant contributor to morbidity and mortality [5]. Therefore, a comprehensive understanding of aortic dissection is crucial for the development of preventive and interventional strategies.

Diabetes, known as diabetes mellitus (DM), is a prevalent global health concern affecting millions worldwide [6]. The four main types of diabetes include type 1 diabetes (T1D), type 2 diabetes (T2D), gestational diabetes related to pregnancy, and other less common forms of the disease [7]. The primary classifications of diabetes typically encompass type 1 diabetes, characterized by insulin dependence resulting from immune-mediated destruction of β cells, and type 2 diabetes, characterized by non-insulin dependence stemming from an insulin secretory defect and insulin resistance [8]. A decade-long study conducted at a single center revealed a lower prevalence of diabetes among patients diagnosed with thoracic aortic dissection [9]. Furthermore, a national case-control study conducted in the United States demonstrated an independent association between diabetes and a reduced likelihood of hospitalization for thoracic aortic aneurysms and dissections, with the severity of diabetic complications influencing this relationship [10]. Diabetes or its pharmacological interventions may confer a protective effect against the occurrence of aortic dissection [11]. However, this evidence stems from observational studies rather than experimental research, precluding definitive causal conclusions and signaling the need for further in-depth investigation.

Ketone bodies, a group of organic chemicals, function as intermediate fat metabolites [12]. It includes β-hydroxybutyrate, acetoacetate, and acetone [13] and are generated during starvation, fasting, or high-fat diet [14]. In addition to their roles in fat metabolism and energy production, ketone bodies offer various health benefits, such as protection against inflammation and oxidative stress [13, 15, 16]. The impact of ketone body metabolism suggests that mild ketosis may have therapeutic potential in a variety of different common and rare disease states [17]. β-hydroxybutyrate, also known as 3-hydroxybutyrate, is a prominent component of ketone bodies circulating in the human body [18] and is present in significant amounts both quantitatively and qualitatively [19]. High levels of 3-hydroxybutyrate have been found to favor improvements in cardiovascular disease [20, 21]. Diabetes is the most common pathologic cause of elevated blood ketones [19]. Given the observed protective effects of diabetes on aortic dissection as well as the recognized cardiovascular benefits of 3-hydroxybutyrate, our study aims to investigate the potential involvement of 3-hydroxybutyrate in mediating the protective effects of diabetes on aortic dissection.

Mendelian randomization, a genetic epidemiological approach, utilizes genetic variation as an instrumental variable to investigate causal relationships between various traits [22]. This method, inspired by Mendel’s laws of inheritance, is akin to a randomized controlled trial [23] and has evolved to include mediation analyses through techniques such as multivariate Mendelian randomization and two-step Mendelian randomization [24]. The utilization of two-step Mendelian randomization for mediation analysis presents a departure from conventional observational mediation analysis techniques, as it offers the ability to consider both the causal effects of the mediator and address potential measurement inaccuracies [25].

In this study, we explored the causal connections between diabetes, 3-hydroxybutyrate, and aortic dissection through two-sample Mendelian randomization (TSMR). Furthermore, we investigated the mediating role of 3-hydroxybutyrate through two-step Mendelian randomization.

## Materials and methods

### Study design

**Fig. 1:**
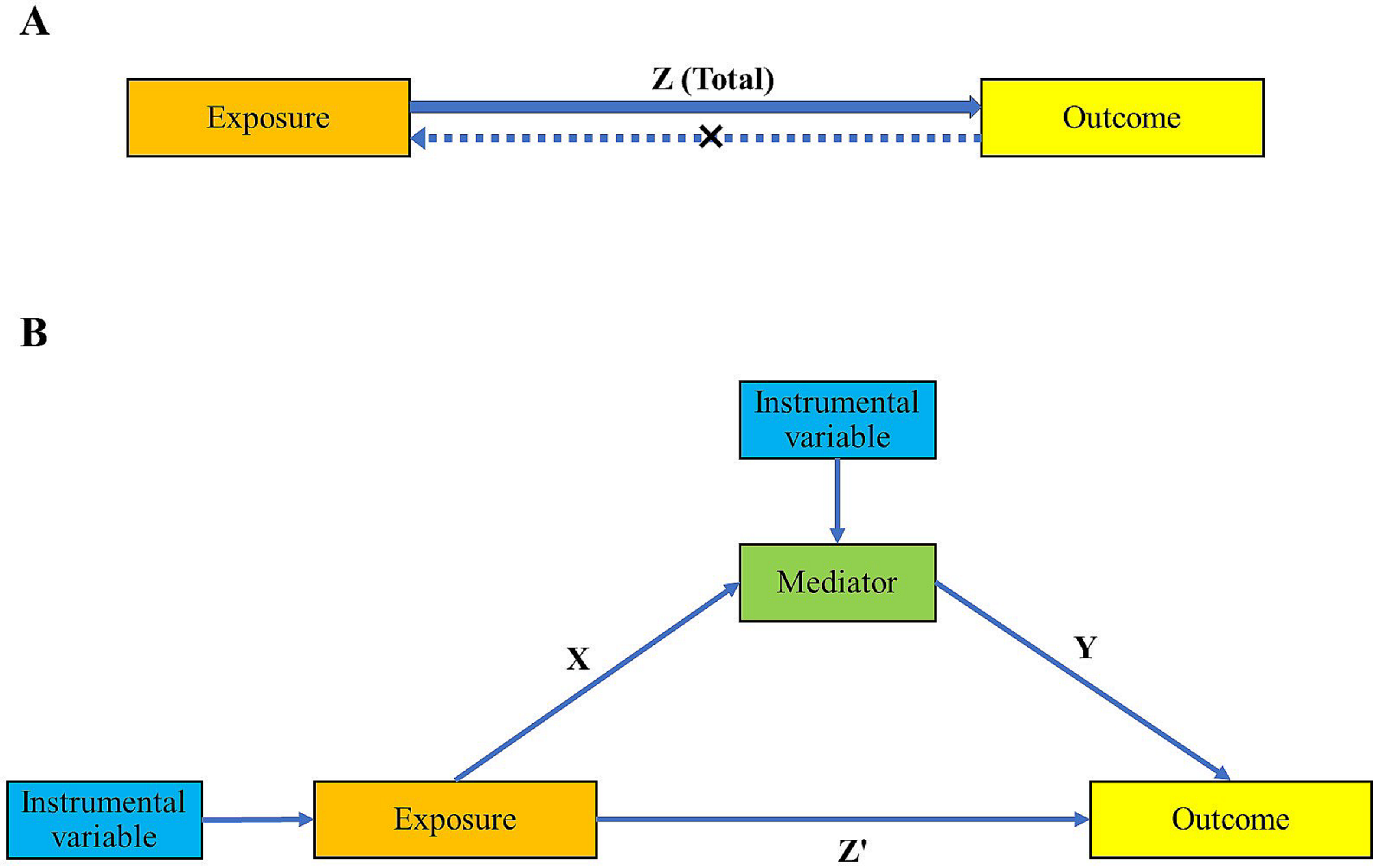
Summary of the mediation MR. Mediated effect = X*Y. Mediated proportion = X*Y/Z

First, a bidirectional two-sample Mendelian randomization was used to investigate the causal association between exposure and outcome (Fig. 1A). In the forward MR analyses, the relationship between exposure and outcome should be correlated. In the reverse MR analyses, the relationship between outcome and exposure should be uncorrelated. Then, a two-step MR design was used to test the causal mediation effect of mediator on the relationship between exposure and outcome (Fig. 1B). In the first step, a two sample MR was used to determine the causal effect of exposure on mediator. If the causal effect was positive, the second step was conducted. In the second step, a two sample MR was used to determine the causal effect of mediator on outcome. Finally, if both of the two steps were positive, the mediation effect was calculated. In our study, the association between Type 1 diabetes and dissection of aorta, the association between Type 2 diabetes and dissection of aorta, and mediation effect of 3-hydroxybutyrate were carried out separately.

### Data sources

Publicly accessible data for Type 1 diabetes, Type 2 diabetes, dissection of aorta and 3-hydroxybutyrate were obtained from genome-wide association studies (GWAS), mainly on European individual characteristics. The GWAS summary statistics for Type 1 diabetes, Type 2 diabetes, and aortic dissection were obtained from FinnGen Release 5, an early personalized medicine initiative that seeks to elucidate genotype-phenotype associations by aggregating and analyzing genomic and health information from participants in Finnish biobanks (https://www.finngen.fi/en). The study population was comprised of 183,185 controls and 5,928 cases for Type 1 diabetes, 184,778 controls and 17,268 cases for Type 2 diabetes, and 206,541 controls and 470 cases for dissection of the aorta. Blood samples from 113,594 UK Biobank participants were utilized to test 3-hydroxybutyrate levels using genome-wide genotyping array [26]. The detail of the traits was showed in Table 1. Due to the fact that the present study drew on publicly available summary-level data from GWAS, no additional ethical approval was required.

**Table 1:** Characteristics of the genome-wide association studies included in the MR study

Trait	Id	Year	Population	Sample size	*n*SNPs
Type 1 diabetes	finn-b-E4_DM1	2021	European	189113	16380008
Type 2 diabetes	finn-b-T2D_WIDE	2021	European	202046	16380418
Dissection of aorta	finn-b-I9_AORTDIS	2021	European	207011	16380411
3-Hydroxybutyrate levels	ebi-a-GCST90092811	2022	European	113594	11590399

### Potential association between type 1 diabetes and dissection of aorta

#### Effect of type 1 diabetes on dissection of aorta

A two sample MR was carried out. Type 1 diabetes was considered as exposure and dissection of aorta was considered as outcome. Single nucleotide polymorphisms (SNPs) representing global human genetic variation were selected as instrumental variables (IVs). SNPs intensely associated with Type 1 diabetes reached a genome-wide significance level (*p* < 5 × 10^ – 8). SNPs were further clumped in the linkage disequilibrium (LD, r2 < 0.001 within 10,000 kb windows). F statistic was used to ensure the strong association between IVs and exposure [27]. SNPs with F statistic greater than 10 were selected. MR pleiotropy residual sum and outlier (MR-PRESSO) test was performed to detect outlier SNPs (Nb Distribution = 1000, Significant Threshold = 0.05) using the MR-PRESSO packages in R software version 4.2.0. Data were harmonized to ensure that the effect of SNPs on exposure and outcome attributed to the same allele before performing the MR analysis. TwoSampleMR packages in R software was used to perform MR analyses. Five MR analysis methods, including MR-Egger, weighted median, inverse-variance weighted (IVW), simple mode, and weighted mode, were analyzed. For sensitivity analysis, heterogeneity was tested for IVW and MR-Egger methods via Cochran’s Q statistics and funnel plots. When p-value for the two methods > 0.05, there was no heterogeneity. Horizontal pleiotropy was tested for MR-Egger and MR-PRESSO methods. When p-value for the two methods > 0.05, there was no horizontal pleiotropy. The leave-one-out analysis was performed to determine if any single SNP was over sensitive and disproportionately responsible for the outcome. We define a result as statistically significant when the p-value for the IVW method of the MR analysis is < 0.05, the five methods of the MR analysis go in the same direction and no horizontal pleiotropy exist.

#### Effect of dissection of aorta on type 1 diabetes

A reverse two sample MR was carried out. Dissection of aorta was considered as exposure and Type 1 diabetes was considered as outcome. The method was the same as described in “Effect of type 1 diabetes on dissection of aorta”.

#### Effect of type 1 diabetes on 3-hydroxybutyrate

A two sample MR was carried out. Type 1 diabetes was considered as exposure and 3-hydroxybutyrate was considered as outcome. The method was the same as described in “Effect of type 1 diabetes on dissection of aorta”.

#### Effect of 3-hydroxybutyrate on dissection of aorta

A two sample MR was carried out. 3-Hydroxybutyrate was considered as exposure and dissection of aorta was considered as outcome. The method was the same as described in “Effect of type 1 diabetes on dissection of aorta”.

#### Mediation effect of 3-hydroxybutyrate

Furthermore, the causal mediation effect of 3-hydroxybutyrate on the relationship between Type 1 diabetes and dissection of aorta was calculated using RMediation packages in R software.

### Potential association between type 2 diabetes and dissection of aorta

#### Effect of type 2 diabetes on dissection of aorta

A two sample MR was carried out. Type 2 diabetes was considered as exposure and dissection of aorta was considered as outcome. The method was the same as described in “Effect of type 1 diabetes on dissection of aorta”.

#### Effect of dissection of aorta on type 2 diabetes

A reverse two sample MR was carried out. Dissection of aorta was considered as exposure and Type 2 diabetes was considered as outcome. The method was the same as described in “Effect of type 1 diabetes on dissection of aorta”.

#### Effect of type 2 diabetes on 3-hydroxybutyrate

A two sample MR was carried out. Type 2 diabetes was considered as exposure and 3-hydroxybutyrate was considered as outcome. The method was the same as described in “Effect of type 1 diabetes on dissection of aorta”.

## Results

### Potential association between type 1 diabetes and dissection of aorta

#### Effect of type 1 diabetes on dissection of aorta

**Fig. 2:**
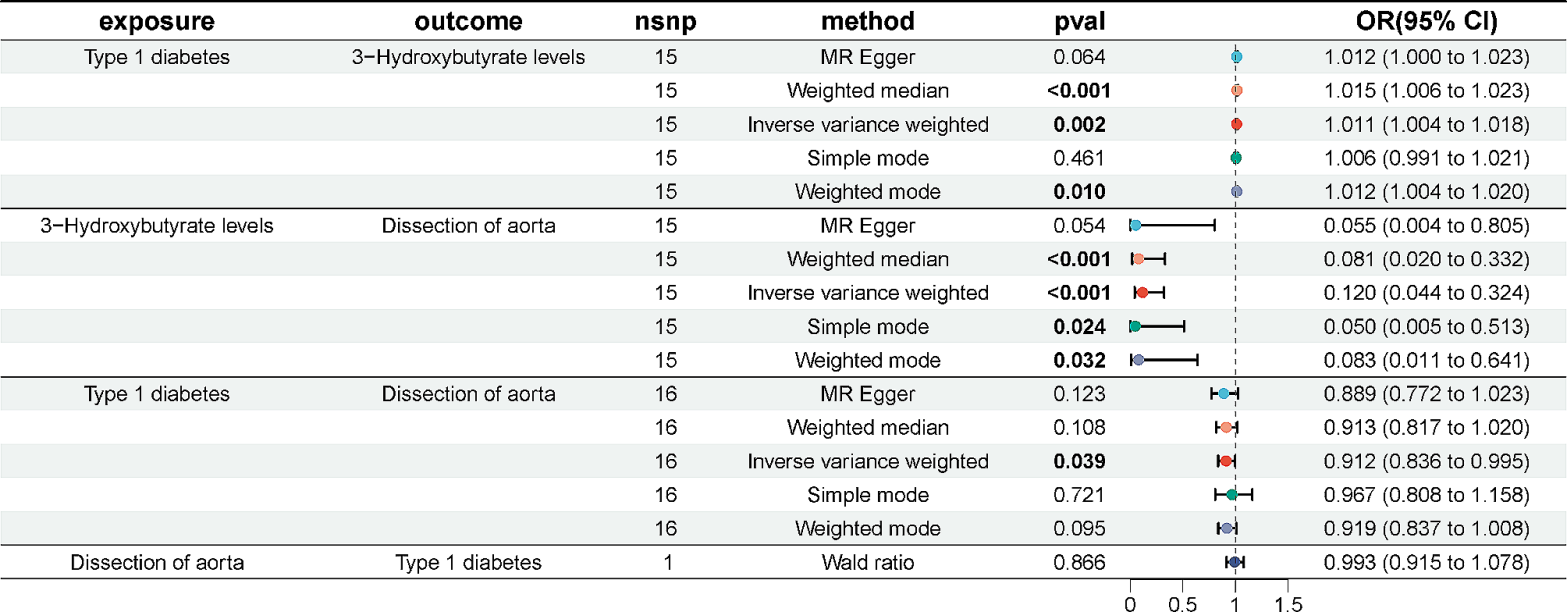
The results of MR analysis to indicate the causal connections between Type 1 diabetes, 3-hydroxybutyrate, and aortic dissection. *OR* odds ratio, *nsnp* number of snp

**Fig. 3:**
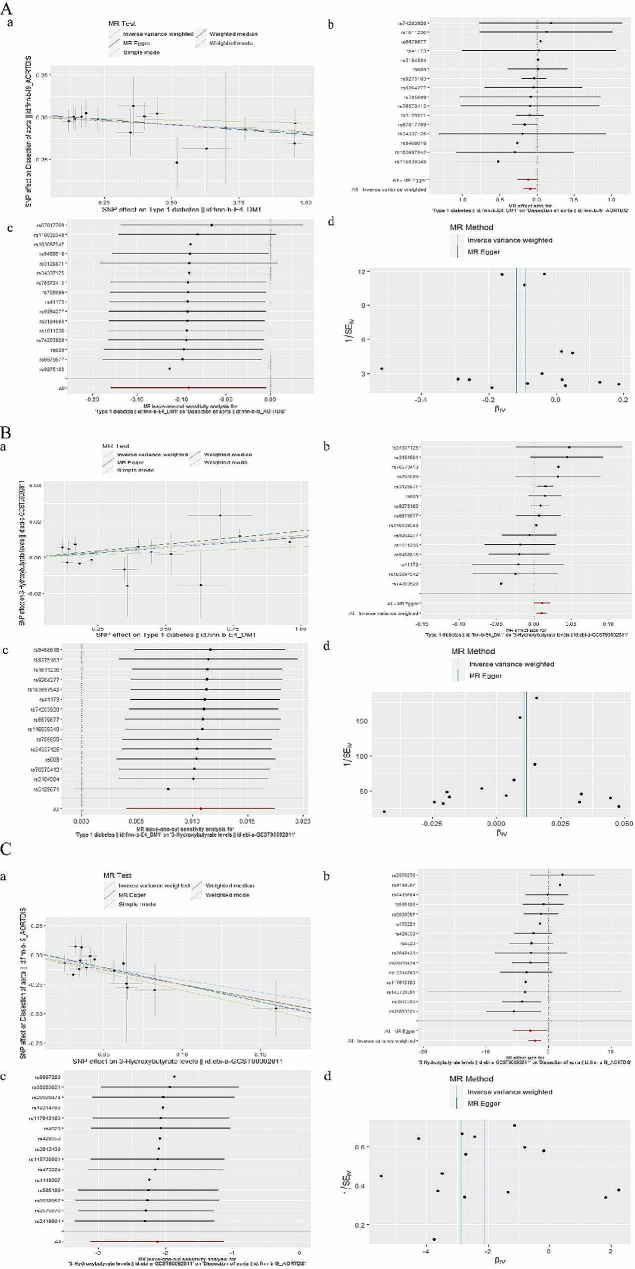
Graphical results of MR analysis to indicate the causal connections between Type 1 diabetes, 3-hydroxybutyrate, and aortic dissection

we selected 17 SNPs from Type 1 diabetes as instrumental variables. F statistic of each SNP was greater than 10. No proxies SNPs were found in outcome. When we harmonize the exposure and outcome SNPs, we found 1 SNP (rs9348894) for being palindromic with intermediate allele frequencies and removed it. The results of the five MR analysis methods were showed in Fig. 2. The IVW method showed that there was a negative association between Type 1 diabetes with the risk of dissection of aorta (OR 0.912, 95% CI 0.836–0.995). The five methods of the MR analysis go in the same direction. These results were also showed in the scatter plot (Fig. 3Aa) and forest plot (Fig. 3Ab). No SNP was over sensitive and disproportionately responsible for the outcome (Fig. 3Ac). No evidence of heterogeneity and pleiotropy between SNPs was observed (Table 2; Fig. 3Ad).

**Table 2:** Tests of heterogeneity and pleiotropy

Exposure	Outcome	Heterogeneity test				Pleiotropy test		
		MR Egger		IVW		MR Egger		MR-PRESSO
		Cochran’s Q	*p* Value	Cochran’s Q	*p* Value	Egger intercept	*p* Value	Global* p* Value
Type 1 diabetes	Dissection of aorta	4.935	0.987	5.145	0.991	0.016	0.654	0.987
Type 1 diabetes	3-Hydroxybutyrate	13.680	0.397	13.707	0.472	-0.0003	0.875	0.538
3-Hydroxybutyrate	Dissection of aorta	11.938	0.533	12.310	0.581	0.037	0.552	0.161
Type 2 diabetes	Dissection of aorta	29.907	0.419	30.122	0.459	0.015	0.651	0.442
Type 2 diabetes	3-Hydroxybutyrate	42.420	0.022	43.499	0.023	-0.002	0.424	0.031

#### Effect of dissection of aorta on type 1 diabetes

we selected 1 SNP from dissection of aorta as instrumental variables. Wald ratio showed that the relationship between dissection of aorta and Type 1 diabetes was uncorrelated (Fig. 2).

#### Effect of type 1 diabetes on 3-hydroxybutyrate

we selected 17 SNPs from Type 1 diabetes as instrumental variables then we found 1 proxies SNP (rs67817769) in outcome aortic dissection and removed it. One outlier SNP (rs9348894) was detected in the MR-PRESSO test and was removed. F statistic of each SNP was greater than 10. The effect of each SNP on the exposure and the outcome attributed to the same allele. The results of the five MR analysis methods were showed in Fig. 2. The IVW method showed that the relationship between Type 1 diabetes and 3-hydroxybutyrate was correlated (OR 1.011, 95% CI 1.004–1.018). The five methods of the MR analysis go in the same direction. These results were also showed in the scatter plot (Fig. 3Ba) and forest plot (Fig. 3Bb). No SNP was over sensitive and disproportionately responsible for the outcome (Fig. 3Bc). No evidence of heterogeneity and pleiotropy between SNPs was observed (Table 2; Fig. 3Bd).

#### Effect of 3-hydroxybutyrate on dissection of aorta

we selected 19 SNPs from 3-hydroxybutyrate as instrumental variables then we found 1 proxies SNP (rs1169297) in outcome aortic dissection and removed it. F statistic of each SNP was greater than 10. When we harmonize the exposure and outcome SNPs, we found 3 SNP (rs10127775, rs12976395, rs2645433) for being palindromic with intermediate allele frequencies and removed them. The results of the five MR analysis methods were showed in Fig. 2. The IVW method showed that the relationship between 3-hydroxybutyrate and dissection of aorta was correlated (OR 0.120, 95% CI 0.044–0.324). The five methods of the MR analysis go in the same direction. These results were also showed in the scatter plot (Fig. 3Ca) and forest plot (Fig. 3Cb). No SNP was over sensitive and disproportionately responsible for the outcome (Fig. 3Cc). No evidence of heterogeneity and pleiotropy between SNPs was observed (Table 2; Fig. 3Cd).

#### Mediation effect of 3-hydroxybutyrate

The results of MR analysis revealed Type 1 diabetes as a protective factor for dissection of aorta. The mediated proportion of 3-hydroxybutyrate on the relationship between Type 1 diabetes and dissection of aorta was 24.80% (95% CI 5.12–44.47%). Details were showed in Table 3.

**Table 3:** Proportion of the effect of type 1 diabetes on dissection of aorta mediated by 3-hydroxybutyrate

Exposure	Mediator	Outcome	Mediated effect	Mediated proportion	*p* Value
Type 1 diabetes	3-Hydroxybutyrate	Dissection of aorta	− 0.02286 (− 0.04099, − 0.00473)	24.80% (5.12%, 44.47%)	0.01348

### Potential association between type 2 diabetes and dissection of aorta

#### Effect of type 2 diabetes on dissection of aorta

**Fig. 4:**
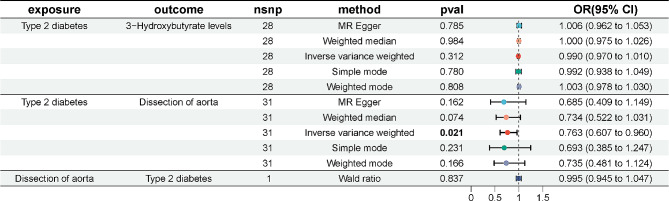
The results of MR analysis to indicate the causal connections between Type 2 diabetes, 3-hydroxybutyrate, and aortic dissection. *OR* odds ratio, *nsnp* number of snp

**Fig. 5:**
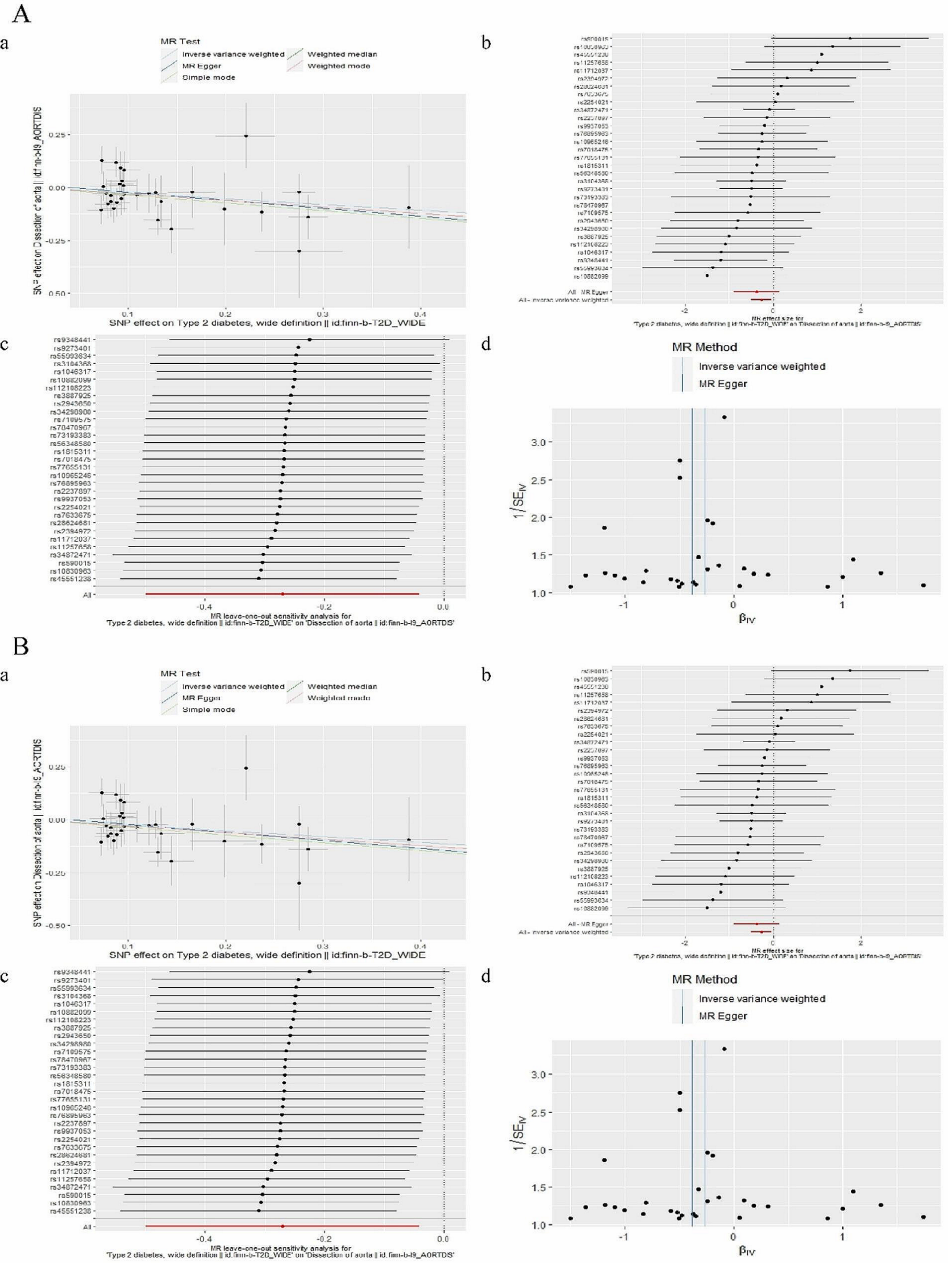
Graphical results of MR analysis to indicate the causal connections between Type 2 diabetes, 3-hydroxybutyrate, and aortic dissection

we selected 31 SNPs from Type 2 diabetes as instrumental variables. F statistic of each SNP was greater than 10. No proxies SNPs were found in outcome. The effect of each SNP on the exposure and the outcome attributed to the same allele. The results of the five MR analysis methods were showed in Fig. 4. The IVW method showed that there was a negative association between Type 2 diabetes with the risk of dissection of aorta (OR 0.763, 95% CI 0.607–0.960). The five methods of the MR analysis go in the same direction. These results were also showed in the scatter plot (Fig. 5Aa) and forest plot (Fig. 5Ab). No SNP was over sensitive and disproportionately responsible for the outcome (Fig. 5Ac). No evidence of heterogeneity and pleiotropy between SNPs was observed (Table 2; Fig. 5Ad).

#### Effect of dissection of aorta on type 2 diabetes

we selected 1 SNP from dissection of aorta as instrumental variables. Wald ratio showed that the relationship between dissection of aorta and Type 2 diabetes was uncorrelated (Fig. 2).

#### Effect of type 2 diabetes on 3-hydroxybutyrate

we selected 31 SNPs from Type 2 diabetes as instrumental variables then we found 1 proxies SNP (rs3104368) in outcome aortic dissection and removed it. Two outlier SNP (rs2943650, rs5634858) was detected in the MR-PRESSO test and was removed. F statistic of each SNP was greater than 10. The effect of each SNP on the exposure and the outcome attributed to the same allele. The results of the five MR analysis methods were showed in Fig. 4. The IVW method showed that the relationship between Type 2 diabetes and 3-hydroxybutyrate was uncorrelated (p-value for the IVW > 0.05). These results were also showed in the scatter plot (Fig. 5Ba) and forest plot (Fig. 5Bb). No SNP was over sensitive and disproportionately responsible for the outcome (Fig. 5Bc). Evidence of heterogeneity and pleiotropy between SNPs were observed (Table 2; Fig. 5Bd). So, 3-hydroxybutyrate does not have causal mediation effect on the relationship between Type 2 diabetes and dissection of aorta.

## Discussion

In this research, we investigated the potential causal relationships between diabetes, 3-hydroxybutyrate, and aortic dissection using a two-sample Mendelian randomization approach. Our findings from the Mendelian analysis indicate that both type 1 and type 2 diabetes exhibit protective effects against the development of aortic dissection. Specifically, individuals with diabetes were shown to have a lower risk of aortic dissection compared to those without diabetes. Furthermore, our results suggest that the protective effects of type 1 diabetes on aortic dissection may be partially mediated by 3-hydroxybutyrate. Nevertheless, our study determined that 3-hydroxybutyrate does not act as a causal mediator in the association between Type 2 diabetes and aortic dissection. Interestingly, our findings suggest that diabetes may actually serve as a protective factor against aortic dissection, aligning with previous real-world observational research. Based on our Mendelian findings, we sought to further explore possible mechanisms in terms of the pathogenesis of aortic dissection, diabetic characteristics, role of ketone bodies and other aspects.

Basically, the aortic wall is composed of three layers: a thin membrane intima facing the blood flow, a thick muscular elastic intima, and an outer fibrous tunica. In cases of acute aortic dissection, a disruption in the intima layer results in the infiltration of blood into the intima-media layer, leading to the formation of an intimal flap that separates the original vessel into true and false lumens. The aorta is susceptible to various influences, including acute occurrences like trauma and hypertensive emergencies, as well as chronic conditions like chronic arterial hypertension, connective tissue disorders, inflammatory vasculitis, and atherosclerosis [28]. The pathological characteristics of aortic dissection have been thoroughly investigated, revealing not only local hemorrhage and chronic inflammatory cell infiltration but also significant disorganization and disruption of elastic fibers within the medial layer of the aorta during dissection [29]. One of the primary histopathologic characteristics of aortic dissection is medial degeneration, marked by the loss of smooth muscle cells and breakdown of the extracellular matrix [30]. A histologic and immunocytochemical analysis of a specimen from an aortic dissection revealed abnormalities in type IV collagen surrounding medial smooth muscle cells. In cases of cystic medial degeneration and mesangial necrosis, the basement membranes of smooth muscle cells were found to be impaired. These findings suggest that alterations in the localized medial basement membrane may play a significant role in the development of aortic dissection [31].

Based on the pathological characteristics of aortic dissection, a number of studies on the pathogenesis of aortic dissection have emerged, mainly involving inflammation, vascular smooth muscle cells (VSMCs), endothelial cells, metabolism and senescence. Research has confirmed that inflammation within the aortic wall contributes to aortic dissection, with some studies indicating that the infiltration of monocytes/macrophages into the aortic wall serves as a primary pathogenic mechanism for the disease [32]. Furthermore, the involvement of B cells and immunoglobulins (specifically IgG) was found to be essential in the inflammatory process leading to aortic dissection pathogenesis. The deposition of fibrinogen, a target of native IgG, was observed to occur prior to the onset of aortic dissection [33]. This abnormal infiltration of pro-inflammatory cells into the aortic wall subsequently triggered apoptosis of vascular smooth muscle cells (VSMCs) and induced the abnormal expression of pro-inflammatory cytokines such as tumor necrosis factor-α (TNF-α), interleukin-1β (IL-1β), and interferon (IF) [34]. The apoptotic process of vascular smooth muscle cells (VSMCs) within the medial layer of the vasculature, as well as the dysfunction of VSMCs, are significant factors in the pathogenesis and progression of aortic dissection [29, 35]. Additionally, the apoptosis of endothelial cells and the death of smooth muscle cells, endothelial cells, and inflammatory cells contribute to the development of aortic dissection [36, 37]. Metabolic abnormalities are observed in both human subjects and animal models of aortic dissection [38]. Recent research indicates that metabolic disorders, specifically affecting amino acid metabolism, glucose metabolism, and lipid metabolism, play a role in the development of aortic aneurysms and dissections by impacting various functional cells within the aorta [39]. Additionally, studies have demonstrated that cellular senescence occurs prior to the onset of aortic dissection and is implicated in its pathogenesis. Furthermore, cellular senescence serves as both a prognostic indicator and a potential target for therapeutic intervention in cases of aortic dissection [40, 41].

After understanding the pathogenesis of aortic dissection, we further explored the possible protective mechanisms of diabetes mellitus against aortic dissection. Hyperglycemia is an important feature of diabetes. It is associated with reduced endothelial neovascularization and decreased inflammatory cell infiltration, which may inhibit aortic dissection progression by reducing VSMC death and extra-cellular matrix (ECM) degradation [10]. In addition, as hyperglycemia influences the VSMC in DM patients, it reduces the risk of aortic dissection by regulating its homeostatic actions in the aortic wall [42]. Hyperglycemia reduces macrophage infiltration within the aortic wall in the aortic dissection model [43]. Several studies have indicated that medications commonly prescribed for diabetes may also offer protective effects against aortic dissection. For example, Metformin has been shown to possess various anti-inflammatory and vasculoprotective properties, such as mitigating aortic inflammation, decreasing extracellular matrix remodeling, and reducing oxidative stress [44, 45]. Furthermore, autonomic remodeling may play a role in lowering the occurrence of STZ-induced aortic dissection in diabetic rats by suppressing matrix metalloproteinase 2 [46].

In our study we found that the protective effects of type 1 diabetes on aortic dissection were partly mediated by 3-hydroxybutyrate, a key constituent of ketone bodies. By gaining a deeper understanding of ketone bodies, we further explored the possible protective mechanisms of 3-hydroxybutyrate against aortic dissection. Ketone bodies have been shown to exert a protective influence in cardiovascular disease by modulating various cellular processes such as gene transcription, inflammation, oxidative stress, endothelial function, cardiac remodeling, and cardiovascular risk factors [47]. Patients with heart failure exhibit elevated levels of ketone bodies in their plasma [48], with the failing heart adapting its energy metabolism to rely more heavily on ketone body utilization [49]. Diabetic patients, akin to those with heart failure, demonstrate a shift in cardiac metabolism characterized by reduced uptake of carbohydrates and increased reliance on 3-hydroxybutyrate and other ketone bodies as alternative energy sources [50]. The failing heart employs 3-hydroxybutyrate as a metabolic stress defense mechanism [51]. Cardiac endothelial cells have the ability to metabolize ketone bodies, leading to enhanced cell proliferation, migration, and vascular sprouting [52]. Furthermore, ketone bodies play a role in regulating endothelial cell homeostasis [53] and can increase antioxidant and anti-inflammatory activities, improve mitochondrial function and growth, facilitate DNA repair, and promote autophagy in response to oxidative stress [54]. Following adherence to a ketogenic diet, there was a notable enhancement in mitochondrial function, a decrease in the expression of apoptotic and inflammatory mediators, and an increase in the activity of neurotrophic factors [55]. Consequently, this dietary approach offers a potential nutritional remedy for alleviating diseases instigated by inflammatory conditions [56]. Additionally, ketone bodies play a crucial role in anti-senescence. In diabetic settings, ketone bodies demonstrate anti-aging properties in podocytes by activating the nuclear factor E2-related factor 2-associated anti-oxidative stress pathway [57]. An increased level of 3-hydroxybutyrate has been suggested as a potential preventive or therapeutic measure for a range of age-related diseases [58]. A recent study has demonstrated that ketosis can effectively prevent the rupture of abdominal aortic aneurysms by modulating the expression of C-C chemokine receptor type 2 (CCR2), inflammatory cytokines, and infiltrating macrophages, while also enhancing the balance of matrix-metalloproteinases (MMPs) [59]. Sodium-glucose cotransporter protein type 2 inhibitors (SGLT2i) were initially formulated as diuretic medications for the management of type 2 diabetes. There exists a conjecture that SGLT2i exhibit the capacity to elevate levels of ketone bodies and free fatty acids, potentially influencing renal and cardiovascular metabolism through the process of ketogenesis [60]. These findings may contribute to a better understanding of the mechanisms underlying the beneficial effects of ketosis.

Combining the pathogenesis of aortic dissection and the role of ketone bodies as described above, we hypothesized the possible links between them. In our study, we found that the protective effect of type 1 diabetes on aortic dissection was partially mediated by 3-hydroxybutyrate, but type 2 diabetes was not 3-hydroxybutyrate mediated. This may be due to the different pathogenesis of type 1 versus type 2 diabetes, as well as differences in ketone body concentrations. The concentration of ketone bodies is higher in type 1 diabetes than in type 2 diabetes. In type 2 diabetes, even when patients are untreated or poorly controlled, serum total ketone body levels do not exceed 2.0 mmol/l. In type 1 diabetes, total ketone bodies are greater than 2 mmol/l [61].

In our study, we applied Mendelian randomization method to illustrate the causal effects of diabetes on the risk of aortic dissection, and investigated the mediating role of 3-hydroxybutyrate in them. In order to establish causality, Mendelian randomization and mediation analyses are dependent on assumptions that are frequently challenging to empirically evaluate [62]. This study was the first MR analysis concerning 3-hydroxybutyrate mediation on diabetes and aortic dissection. Unlike randomized controlled trials, Mendelian randomization effectively mitigates potential confounding variables by virtue of the random allocation of alleles at conception. Furthermore, the study was limited to a European cohort to reduce the impact of population stratification. Our findings did not indicate any heterogeneity or pleiotropy within the study population. During the mediation analysis phase, a statistically significant mediated proportion of 24.80% was observed with a p-value of less than 0.05. Despite the validity of our Mendelian randomization analyses, limitations exist within our study, primarily due to its focus on a European population. It is imperative to confirm these findings in other populations. In the mediation analysis, a two-step Mendelian randomization approach was utilized, excluding the use of multivariate Mendelian randomization. This omission resulted in the exclusion of certain genetic variants potentially linked to lipid levels, blood pressure, inflammatory markers, insulin resistance, and susceptibility to coronary heart disease, thereby diminishing the robustness of the analysis, thus serving as a limitation of the study. Reports indicate variations in ketone body concentrations between individuals with type 1 and type 2 diabetes, prompting further investigation into potential implications of these differences. However, due to the pooling of online GWAS data, we were unable to stratify ketone body concentrations for each type of diabetes, thus presenting a limitation in our study. While Mendelian randomization revealed a mediating effect of 3-hydroxybutyrate, additional mechanistic studies are warranted.

## Conclusions

In summary, our Mendelian randomization analysis demonstrated that diabetes may confer a protective effect against aortic dissection. Specifically, type 1 diabetes was found to exhibit a partial protective effect on aortic dissection through mediation by 3-hydroxybutyrate, while type 2 diabetes did not show this mediation. The protective association highlighted by our findings suggests that interventions such as a ketogenic diet or exogenous ketone supplementation could be explored as potential preventive measures for aortic dissection in future clinical investigations. Further mechanisms of diabetic protection against aortic dissection need to continue to be explored.

## Data Availability

No datasets were generated or analysed during the current study.

## Abbreviations

GWAS: Genome-wide association studies
MR: Mendelian randomization
DM: Diabetes mellitus
T1D: Type 1 diabetes
T2D: Type 2 diabetes
TSMR: Two-sample Mendelian randomization
SNPs: Single nucleotide polymorphisms
IVs: Instrumental variables
LD: Linkage disequilibrium
MR-PRESSO: MR pleiotropy residual sum and outlier
IVW: Inverse-variance weighted
VSMCs: Vascular smooth muscle cells
TNF-α: Tumor necrosis factor-α
IL-1β: Interleukin-1β
IF: Interferon
ECM: Extra-cellular matrix
CCR2: C-C chemokine receptor type 2
MMPs: Matrix-metalloproteinases
SGLT2i: Sodium-glucose cotransporter protein type 2 inhibitors
